# Primary intestinal lymphangiectasia presenting as limb hemihyperplasia: a case report and literature review

**DOI:** 10.1186/s12876-021-01813-6

**Published:** 2021-05-18

**Authors:** Ammar A. Khayat

**Affiliations:** Department of Pediatrics, Gastroenterology Unit, Department of Pediatrics, Faculty of Medicine, Umm AL Qura University, King Abdulaziz University, 24381 Al-Abdiyyah, Makkah, Saudi Arabia

**Keywords:** Lymphedema, Intestinal lymphangiectasis, Primary intestinal lymphangiectasia, Saudi Arabia, Hemihyperplasia, Case report

## Abstract

**Background:**

Primary intestinal lymphangiectasia is an exceedingly rare disorder. Epidemiology is unknown. It usually presents with lower extremity swelling, diarrhea, ascites, and protein-losing enteropathy. Since the pathogenesis of edema is usually due to hypoalbuminemia; both extremities are typically involved. The edema can rarely be due to abnormal lymphatic circulation, causing lymphedema, which usually involves both extremities as well. Diagnosis is made by the constellation of clinical, biochemical, endoscopic, and histological findings. Treatment involves dietary modification, to reduce lymphatic dilation in response to dietary fat. Other pharmacologic (e.g., octreotide) and replacement measures may be indicated as well. The most serious long-term complication is intestinal lymphoma. Herein is a case of Primary intestinal lymphangiectasia presenting with unilateral lower limb swelling.

**Case presentation:**

A 4-year-old boy presents with left foot swelling since the age of 4 months, in addition to intermittent diarrhea, and abdominal swelling. The foot swelling had been evaluated by different health care professionals in the past, and was mislabeled as either cellulitis, or congenital hemihyperplasia. Physical examination revealed mild ascites, and a non-pitting foot edema with a positive Stemmer’s sign (lymphedema). Blood work revealed hypoalbuminemia (albumin 2 g/dl), and hypogammaglobulinemia. Endoscopy showed dilated lacteals throughout the duodenum. Histopathologic examination revealed massively dilated lamina propria lymphatics in the duodenal biopsies. The patient was diagnosed with primary intestinal lymphangiectasia. He was treated with high-protein and low-fat diet, and supplemental formula high in medium chain triglycerides. On follow-up, the patient’s diarrhea completely resolved, and his ascites and edema improved significantly.

**Conclusions:**

The presence of unilateral lower limb edema should not preclude the diagnosis of systemic disorders, and a high index of suspicion is required in atypical presentations. A good knowledge about Primary intestinal lymphangiectasia manifestations, and physical examination skills to differentiate edema or lymphedema from tissue overgrowth can significantly aid in the diagnosis.

## Background

Primary intestinal lymphangiectasia (PIL) was first described by Waldman et al. in 1961, when they noticed an association between hypoproteinemia and gut protein loss [1]. Since then, sporadic cases from around the world have been reported on this rare disease [2, 3]. Prevalence is unknown, with most studies being limited to case reports and series [4]. The majority of patients present in the first 3 years of life [4, 5]. The most common reported presentation is bilateral lower limb edema, with or without ascites, and often with diarrhea [4, 5]. PIL typically responds well to dietary modification [4, 5]. Herein is a case of PIL presenting with unilateral edema, initially mislabeled as limb hemihyperplasia (hemihypertrophy), and ascites.

## Case presentation

A 4-year-old boy with no known medical condition presents to an outpatient clinic with history of left foot swelling and diarrhea. The swelling was noted by parents at the age of 4 months, which had been evaluated by medical professionals and attributed to a local infection according to parents. It had never disappeared completely, despite a waxing and waning course, which led parents to believe it was a congenital condition. During the course, a physician mislabeled the swelling as hemihyperplasia (hemihypertrophy), due to the stark difference in the size of the feet. More recently, the patient started complaining of intermittent watery to semi-formed stools, 3–6 times per day, that were aggravated by fatty diet, and were occasionally difficult to flush. He was noted to have poor subcutaneous fat in his face and arms but did not lose weight. Family history was negative for lymphedema, or any other genetic disorder.

**Fig. 1:**
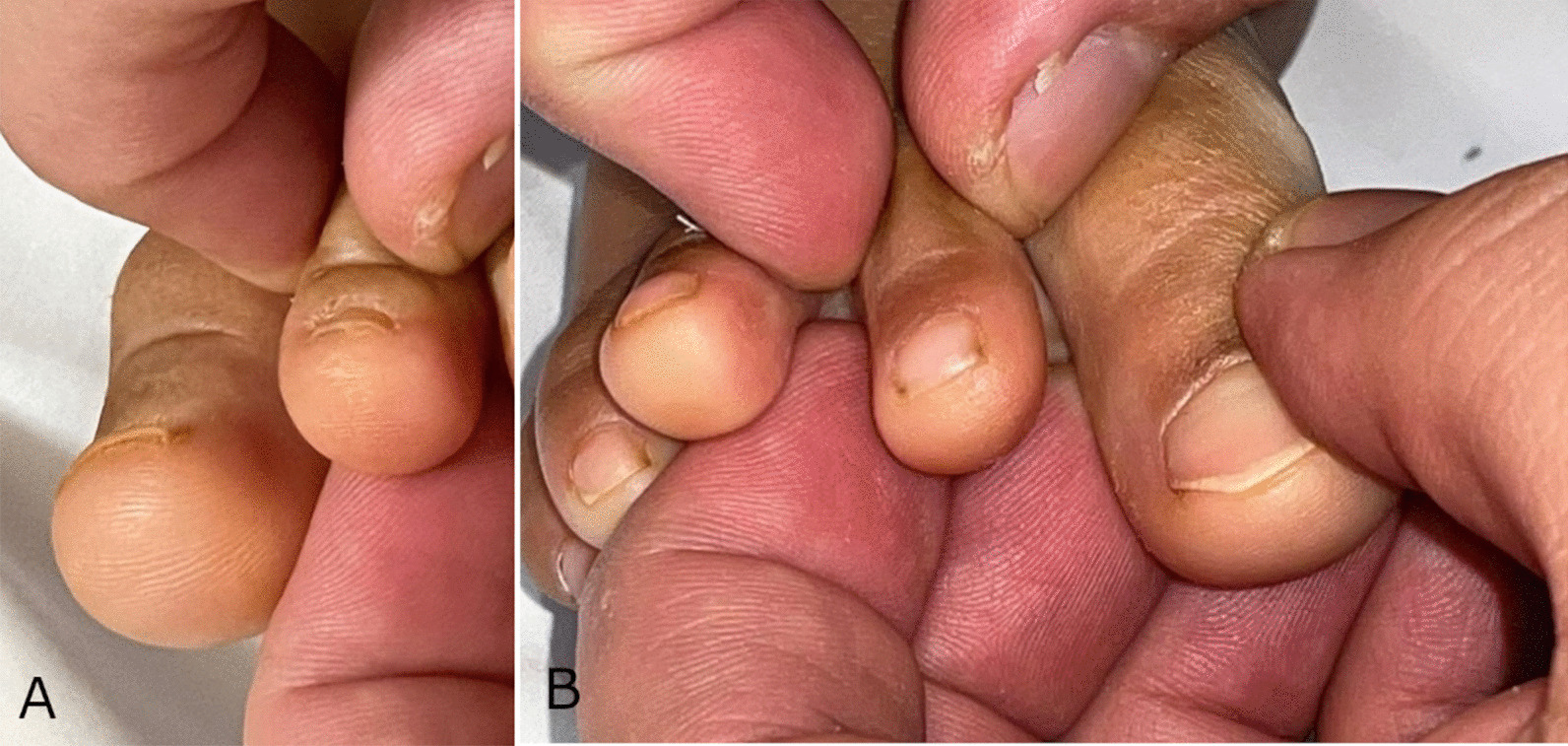
**a** and **b** Photographic images of the patient’s left foot (**a**), showing a positive Stemmer’s sign: the skin on the dorsum of the second toe cannot be easily pinched, compared to the right foot (**b**)

On examination, he was vitally stable. He looked undernourished even though his weight was on the 90th centile, likely due to ascites. Abdominal examination revealed mild ascites, normal bowel sounds, and no organomegaly. Cardiac and respiratory examinations were unremarkable. His right foot showed almost no edema. Left foot examination revealed a significantly swollen dorsal aspect of the foot and toes confirming presence of edema up to the level of his ankle. The edema was minimally pitting, with a positive Stemmer’s sign (Fig. 1). Ankle joint examination was otherwise unremarkable.

**Fig. 2:**
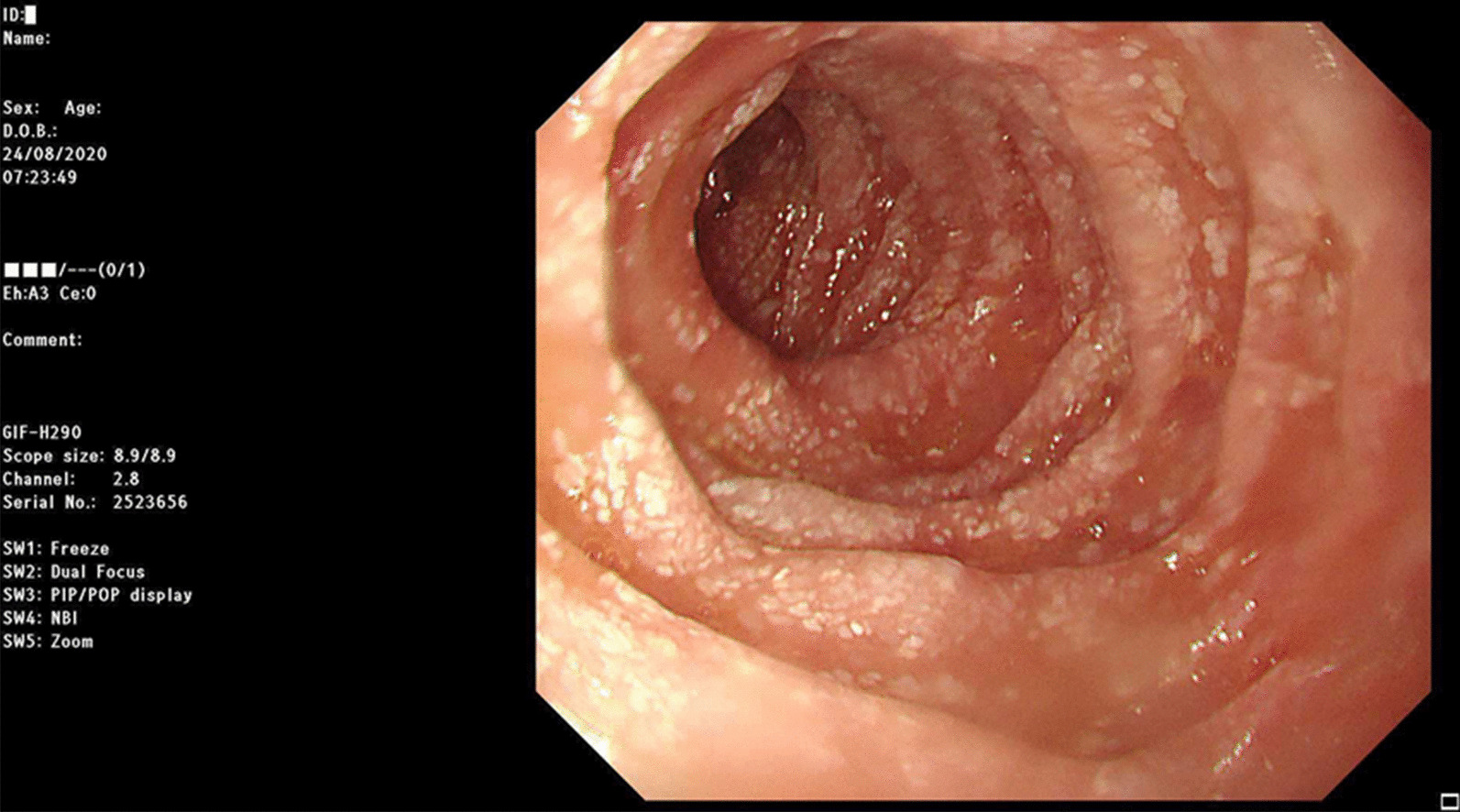
Endoscopic image of the duodenum at the level of the second part, showing the mucosa completely covered with white spots or dilated “lacteals”

**Fig. 3:**
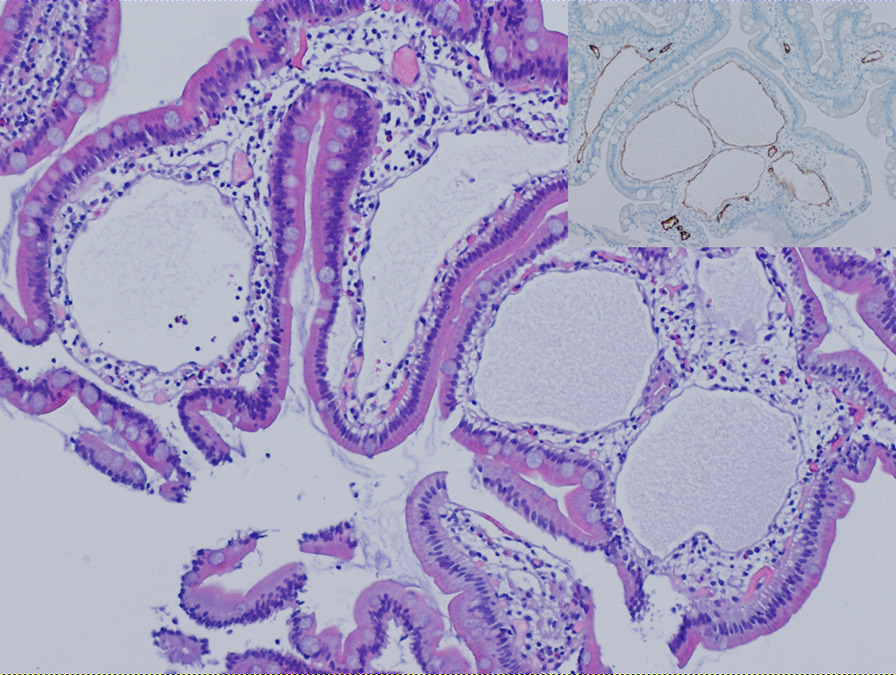
Hematoxylin and Eosin stain of duodenal mucosal biopsy at 10X magnification, showing massively dilated lymphatic vessels in lamina propria, glandular and surface epithelium are unremarkable otherwise. Podoplanin stain highlighting the lymphatic vascular endothelium of duodenal mucosal biopsy at 10X magnification is shown in inset, again redemonstrating the massive dilation

Laboratory workup showed a low albumin at 2 g/dl (normal range 3.5–5.5 g/dl), total protein at 2.9 g/dl (normal range 6–8 g/dl), total IgG at 147 mg/dl (normal range 565–1765 mg/dl), IgM at 16 mg/dl (normal range 55–375 mg/dl), and IgA at 30 mg/dl (normal range 85–385 mg/dl). Other tests were essentially within normal limits including a negative urine protein, normal liver enzyme levels, and a negative tissue transglutaminase IgA antibody. Upper and lower gastrointestinal endoscopies were performed and showed the classic appearance of white spots or dilated “lacteals” in the duodenum (Fig. 2). The colon showed lymphoid nodular hyperplasia. Otherwise, endoscopy was unremarkable. Histopathologic examination showed a remarkably dilated lamina propria lymphatic vessels in the duodenum (Fig. 3). Otherwise, no other significant abnormalities were observed. Lymphoscintigraphy of his left foot showed failure of progression to the proximal lymph nodes confirming the presence of massively dilated lymphatics.

The diagnosis of primary intestinal lymphangiectasia was made based on the constellation of clinical, biochemical, endoscopic, and histological findings.

The patient was started on high protein, minimal fat diet, along with supplemental high medium chain triglyceride (MCT) formula.

**Fig. 4:**
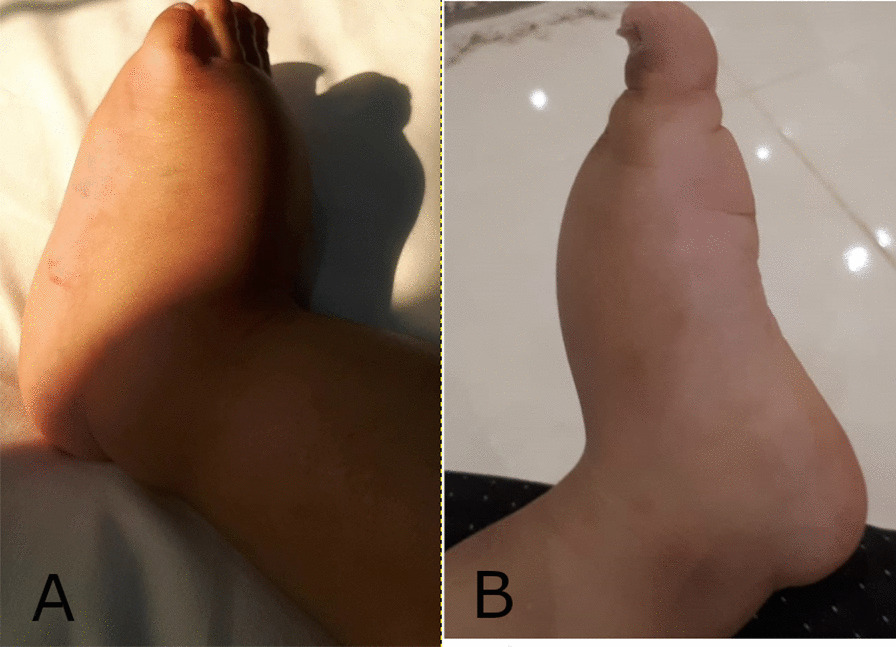
A and B: Photographic images of the patient’s left foot showing the edema prior to dietary modification (**a**) and the significant improvement after dietary modification (**b**)

On follow up, his weight remained on the 90th centile, however, his mid-arm circumference was on the 75th centile. His bowel movement frequency, and consistency improved immediately, and so did his subcutaneous fat eventually. His ascites resolved completely, and his left leg swelling was significantly improving as well (Fig. 4), with persistence of Stemmer’s sign however. His Albumin and total protein levels improved as well.

## Discussion and conclusion

Unilateral limb swelling has a wide array of differential diagnoses, such as different types of lymphedema (primary and secondary) [6, 7]. True Hemihyperplasia is seen in syndromes such as Beckwith-Weidemann syndrome, or could be an isolated disorder [8]. Hemihyperplasia differs from lymphedema where there is a true tissue overgrowth in the former, whereas only accumulation of excess interstitial fluid in the latter [7–9].

primary lymphedema without systemic manifestations or visceral involvement (i.e., PIL, or chylous effusions) also occurs in a familial pattern and is often due to a genetic disorder [7]. The onset can be congenital, or present later in life, and can be localized to the lower extremities or other body parts, including upper extremities and face [7]. The edema can be unilateral, but more commonly bilateral, as seen in Meige, and Melroy syndromes [7, 10]. PIL in comparison, commonly presents as an isolated, or non-familial disorder [5, 11]. Rarely, it may occur in a familial or a syndromic pattern, where there is a widespread lymphatic malformations or dysplasia [5, 11]. Examples include Turner, Noonan, and Hennekam syndromes [5, 12]. The lymphedema associated with PIL (whether syndromic or isolated) is typically bilateral [4, 5].

Hypoalbuminemia is another mechanism of edema in PIL, which in fact, is more common than lymphedema [1, 5]. The underlying pathophysiology is lymph loss in bowel lumen, from excessive dilation and eventually rupture of the already ectatic lymphatic vessels of small bowels [1, 5]. Intestinal lymph is rich in long chain triglycerides, lymphocytes, and proteins [13]. The result of excessive lymph loss is the characteristic steatorrhea, lymphopenia, edema, and hypoproteinemia including hypogammaglobulinemia [1, 4, 5, 13]. Interestingly, the case herein predominantly had a combined pitting and non-pitting unilateral edema, despite presenting with systemic manifestations and visceral involvement (PIL and ascites).

The most common clinical presentation in PIL includes bilateral lower extremity edema and intermittent or chronic diarrhea [4, 5, 14]. As discussed earlier, The edema is mainly pitting; due to hypoalbuminemia, but could rarely be non-pitting as well, when it is due to lymphedema [5, 11, 15, 16]. It is oftentimes difficult to distinguish them, especially when both are present [5]. Stemmer’s sign can be helpful in such cases, where a positive sign (the inability to pinch the skin at the dorsum of the second toe) indicates lymphedema, rather than pitting edema [5, 17]. Other clinical manifestations of PIL including abdominal mass, chylous effusion (ascitic, pleural, or pericardial), generalized anasarca, and intestinal mechanical obstruction have been rarely reported [5, 18, 19].

PIL diagnosis is usually made by endoscopic, and histopathologic findings of the small intestines, showing the typical dilated lymphatic vessels in the lamina propria and submucosa [4, 5]. Limb lymphatic vessels can be examined by various radiographic techniques, which can aid in the diagnosis, and to rule out secondary causes of limb lymphedema or chylous effusions [5, 20]. Examples include lymphoscintigraphy, lymphangiography, and magnetic resonance lymphangiography [5, 20].

Treatment focuses mainly on dietary modification [4, 5, 11]. dietary fat induces dilation of lymphatics even in the normal intestines. Therefore, avoidance of fat decreases excessive dilation and risk of rupture in lymphangiectasia [5, 13]. High MCT containing formulas, and fat restriction along with high protein diet are the cornerstones of treatment for infants, and older children respectively [4, 5, 11]. MCTs are absorbed directly into the portal venous circulation and do not require lymphatic flow for absorption [21]. Octreotide use for the treatment of chylous effusions has been described in the literature [22]. Its effect on the intestines is unclear, but it is hypothesized that it decreases triglycerides absorption, and induces splanchnic vasoconstriction [5]. Albumin and intravenous immunoglobulin (IVIG) infusions are frequently indicated as replacement therapies, depending on the levels and clinical scenarios [5]. Other treatment measures include managing any nutritional deficiencies, specifically fat-soluble vitamins [5, 23].

The most serious but rare long-term complication reported in PIL is intestinal B cell lymphoma [4, 24]. Other complications include nutritional deficiencies, lower extremity cellulitis, and the effects of chronic foot swelling on quality of life [5, 23].

In conclusion, PIL is a rare disorder that typically presents with protein-losing enteropathy, diarrhea, and bilateral lower limb edema. Nonetheless, unilateral lower limb edema (non-pitting more so than pitting) should not preclude the diagnosis of a systemic disorder, and a high index of suspicion is required in atypical presentations. A good knowledge about PIL, and physical examination skills to differentiate edema or lymphedema from tissue overgrowth can significantly aid in the diagnosis. PIL responds well to dietary modification, but needs long-term monitoring into adulthood, for potentially serious complications.

## Data Availability

Not applicable.

## Abbreviations

IVIG: Intravenous immunoglobulin.
MCT: Medium chain triglycerides.
PIL: Primary intestinal lymphangiectasia.
